# Anti-Inflammatory and Antipruritic Effects of Luteolin from Perilla (*P. frutescens* L.) Leaves

**DOI:** 10.3390/molecules19066941

**Published:** 2014-05-27

**Authors:** In Hwa Jeon, Hyeon Soo Kim, Hyun Ju Kang, Hyun-Seo Lee, Seung Il Jeong, Sang Jun Kim, Seon Il Jang

**Affiliations:** 1 Ato Q&A Corporation, Jeonju 560-759, Korea; E-Mails: inflowerj@hanmail.net (I.H.J.); dkgk0608@naver.com (H.J.K.); 2 Department of Health & Science, Jeonju University, Jeonju 560-759, Korea; E-Mails: badaloves@naver.com (H.S.K.); jkll4903@hanmail.net (H.-S.L.); 3 Jeonju Biomaterials Institute, Jeonju 561-360, Korea; E-Mails: siunite@hanmail.net (S.I.J.); biomale@hanmail.net (S.J.K.)

**Keywords:** perilla leaves, luteolin, mast cells, scratching behavior, anti-inflammation, antipruritus

## Abstract

Perilla (*Perilla frutescens* L.) leaves have shown therapeutic efficacy in the treatment of inflammatory disorders, allergies, bronchial asthma, and systemic damage due to free radicals. In the present study we analyzed the active constituents in perilla leaves using high-performance liquid chromatography (HPLC) and isolated luteolin, a polyphenolic flavonoid. We investigated the anti-inflammatory and antipruritic properties of luteolin. Luteolin inhibited the secretion of inflammatory cytokines such as interleukin-1β (IL-1 β) and tumor necrosis factor-α (TNF-α) from human mast cells (HMC-1) stimulated with phorbol myristate acetate plus calcium ionophore A23187 in a dose-dependent manner. Luteolin also significantly reduced the histamine release from rat peritoneal mast cells stimulated by compound 48/80, a potent histamine liberator. Furthermore, the administration of luteolin markedly inhibited the scratching behavior and vascular permeability induced by pruritogens, such as compound 48/80 or serotonin, in ICR mice. These results suggested that luteolin has potential as a therapeutic agent against inflammation and itch-related skin diseases.

## 1. Introduction

Inflammation is a protective aspect of the body’s response to injury or infection and induces release of various chemicals that stimulate nerve endings. The main symptoms of inflammation include redness, swelling, heat and pain. In chronic inflammatory diseases caused by persistent inflammation, a progressive shift of the cell type at the area of inflammation occurs, causing considerable tissue damage [1]. During inflammation, the inflammatory region is infiltrated with mononuclear cells such as monocytes, macrophages and lymphocytes, producing a wide range of inflammatory mediators including pro-inflammatory cytokines [2].

Mast cells have been recognized for their participation in allergic reactions as well as in inflammatory processes based on their ability to secrete numerous cytokines, chemokines and preformed mediators such as histamine [3,4]. When mast cells are activated by allergens bound to IgE-FcεRI on the cell surface, they release histamine and mast cell granule proteins as well as a wide variety of pro-inflammatory mediators such as interleukin (IL)-1, IL-6, IL-8, and tumor necrosis factor (TNF)-α, which may exacerbate allergic diseases [5,6]. These mediators affect nerve, muscle, and endothelial cells and induce itching, contraction, vasodilation and edema. Notably, modulation of mast cell cytokine production can provide a useful therapeutic strategy for allergic inflammatory diseases, which include atopic dermatitis, allergic asthma and allergic rhinitis [7].

Pruritus or itch, which is defined as an unpleasant cutaneous sensation associated with the immediate desire to scratch, is the diagnostic hallmark of dermatological diseases such as atopic dermatitis [8,9]. Itch can lead to repetitive scratching until bleeding. The severe scratching causes skin lesions, and consequently aggravates the skin disease. Therefore, inhibition of the itch that provokes scratching would improve the patient’s quality of life and enable treatment of the original disease [10]. Numerous studies have focused on identification of compounds in plant extracts capable of treating itch-related skin diseases [11].

Perilla (*Perilla frutescens* L.) is an annual herb of the Lamiaceae family mainly used in Korea, Japan and China and with many beneficial properties [12]. Perilla leaves have long been used to treat various diseases, including depression, tumors, bacterial and fungal infections, allergies and some intestinal disorders. They also inhibit Th2-cytokine production, anti-DNP IgE production and IgA nephropathy [13,14]. The main active constituents of perilla leaves include flavonoids, saponins, polysaccharides, amino acids and trace elements [12,14,15]. The flavonoids existing in many types of plant have been reported to exert anti-inflammatory and anti-allergic activities by suppressing the proliferation and activity of lymphocytes [16,17].

In the present study we focused on luteolin, a flavonoid extracted from perilla leaves and investigated its anti-inflammatory and antipruritic effects on human mast cells (HMC-1), rat peritoneal mast cells (RPMCs) and ICR mice.

## 2. Results and Discussion

### 2.1. The Chemical Structure and Effect of Luteolin on Cell Survival

**Figure 1:**
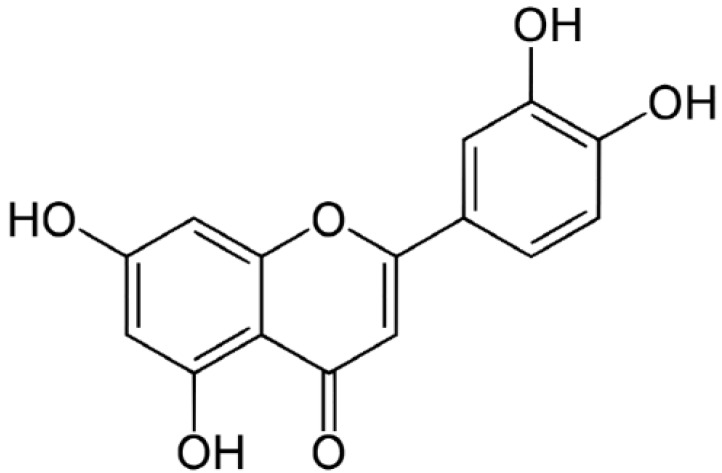
The structure of luteolin isolated from perilla (*P. frutescens*) leaves.

**Figure 2:**
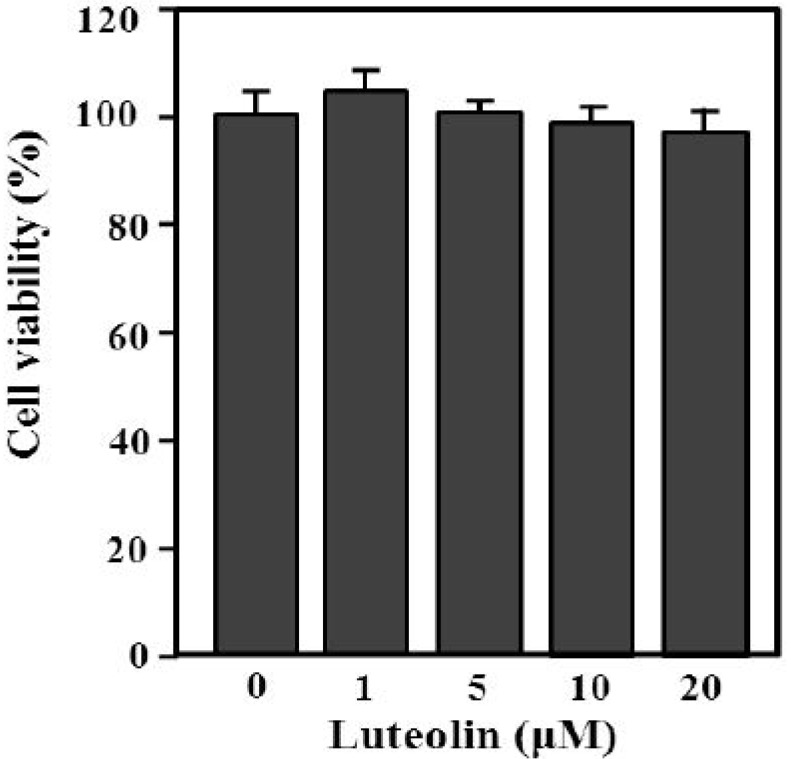
Effect of luteolin on HMC-1 cell viability. Cells (5 × 10^5^) were treated with 1–20 μM luteolin and then incubated at 37 °C for 12 h. Cell viability was determined by MTT assay. Values are means ± standard deviation (SD) of duplicate determinations from three independent experiments.

The content of luteolin isolated from perilla leaves by HPLC was 22.58 μg/g. The chemical structure of luteolin, a plant flavonoid, is shown in Figure 1. Luteolin is a pharmacologically active agent isolated from several herbal species such as fruits, vegetables and medicinal plants [18,19]. Flavonoids play an important role in protecting plant cells from microorganisms, insects and ultraviolet (UV) radiation. They also exhibit cancer preventive, antioxidative, anti-allergic and anti-inflammatory activities in cells [10,20]. Recently, luteolin isolated from the *Lonicera japonica* flowers was shown to inhibit pro-inflammatory cytokine and inflammatory mediator production by HMC-1 cells activated with PMA plus A23187, and suppress inflammation-associated gene expression by blocking the NF-κB pathway [21,22,23]. These reports showed that plants rich in luteolin have been used to treat diseases such as inflammation, allergy and cancer since ancient times. However, studies of the anti-inflammatory effects of luteolin isolated from perilla leaves are lacking. Therefore, we examined whether luteolin isolated from perilla leaves has anti-inflammatory and antipruritic effects in HMC-1 cells, RPMCs, and ICR mice. First, we examined the cytotoxicity of luteolin against HMC-1 cells by MTT assay. As shown in Figure 2, luteolin did not affect cell viability.

### 2.2. Inhibition of TNF-α and IL-1β Production by Luteolin

Mast cells are one of the major immune effector cells. Activated mast cells release pro-inflammatory cytokines, such as TNF-α, IL-1β, IL-6 and IL-8, and inflammatory mediators, including histamine, leukotrienes, serotonin, prostaglandin (PG)E_2_, and PGD_2_ [5,7,24]. TNF-α and IL-1β cytokines are released in a coordinated network and play an important role in chronic inflammation. TNF-α is preformed and stored in granules of mast cells or newly synthesized following mast cell activation; it is a multifunctional cytokine and an important mediator of the immune and inflammatory responses. TNF-α is an autocrine stimulator as well as a potent inducer of other inflammatory cytokines, including IL-1β, IL-6, and IL-8 [6]. IL-1β is a pro-inflammatory cytokine and a potent mediator of inflammatory processes [25].

**Figure 3:**
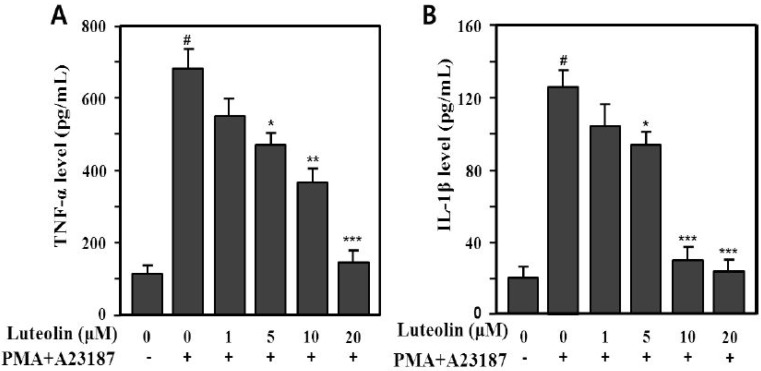
Inhibitory effect of luteolin on PMA plus A23187-induced TNF-α (**A**)- and IL-1β (**B**) production in HMC-1 cells. Cells (5 × 10^5^) were pretreated with 1–20 μM luteolin for 30 min prior to stimulation with or without 25 nM PMA plus 1 μM A23187 and then incubated at 37 °C for 12 h. Cytokine levels were determined by enzyme-linked immunosorbent assay (ELISA). Values are means ± SD of duplicate determinations from three independent experiments. ^#^ *p* < 0.001 *versus* the non-treated control group; * *p* < 0.05, ** *p* < 0.05, *** *p* < 0.01 *versu* PMA plus A23187 alone group.

Therefore, we examined whether luteolin could modulate production of pro-inflammatory cytokines, such as TNF-α and IL-1β, induced by PMA plus A23187 in HMC-1 cells. The supernatants were analyzed using an enzyme-linked immunosorbent assay (ELISA) for TNF-α and IL-1β. PMA plus A23187 significantly increased TNF-α (688.5 ± 47.3 pg/mL) and IL-1β (129.2 ± 9.1 pg/mL) production compared with the medium control (TNF-α: 110.5 ± 21.3 pg/mL, IL-1β: 19.8 ± 5.11 pg/mL) in HMC-1 cells (*p* < 0.001). We also assessed the effect of luteolin (1–20 μM) on PMA plus A23187-induced TNF-α and IL-1β production. The increased production of TNF-α and IL-1β induced by PMA plus A23187 was significantly inhibited by 5–20 μM luteolin (Figure 3). The inhibition rates were 31.9%–76.8% (TNF-α) and 27.3%–81.2% (IL-1β) at 5–20 μM luteolin.

### 2.3. Inhibition of Histamine Release by Luteolin

**Figure 4:**
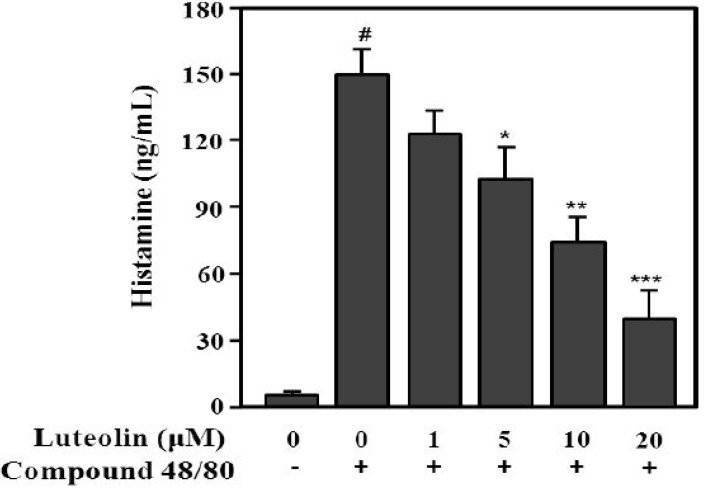
Inhibitory effect of luteolin on histamine released from RPMCs activated with compound 48/80. RPMCs (5 × 10^5^/mL) were pre-treated with or without luteolin at the indicated concentrations for 2 h, and then stimulated with or without compound 48/80 (5 μg/mL). Histamine levels in RPMC supernatants were determined by ELISA. Values are means ± SD of three independent experiments. ^#^ *p* < 0.001 *versus* the non-treated control group; * *p* < 0.05, ** *p* < 0.05, *** *p* < 0.01 *versus* compound 48/80 alone group.

Compound 48/80 increases the permeability of the lipid bilayer by causing a perturbation in the membrane [26]. Additionally, compound 48/80 can activate the release of histamine from mast cells [27]. Next, to evaluate the inhibitory effect of luteolin on histamine release, RPMCs were treated with compound 48/80. As shown in Figure 4, compound 48/80 significantly enhanced histamine release (148.7 ± 14.9 ng/mL) compared with the control (6.7 ± 2.5 ng/mL; *p* < 0.001). Luteolin significantly inhibited the compound 48/80-induced histamine release at 5, 10, and 20 μM in a dose-dependent manner (*p* < 0.05, *p* < 0.01 and *p* < 0.001, respectively).

### 2.4. Effects of Luteolin on Scratching Behavior and Vascular Permeability

**Figure 5:**
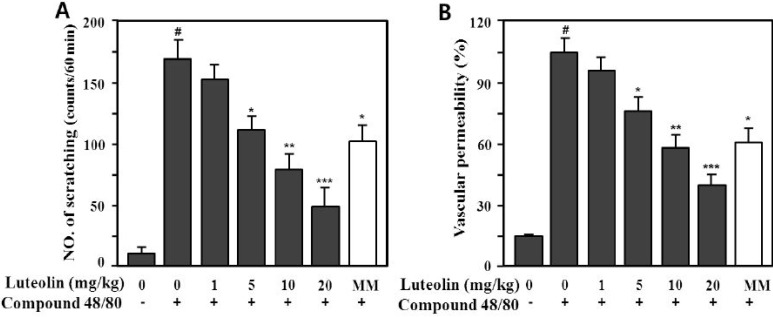
Effect of luteolin on compound 48/80-induced scratching behavior (**A**) and vascular permeability (**B**) in mice. Scratching behavior was counted for 60 min after intradermal injection of compound 48/80 (50 μg/site). Luteolin (1–20 mg/kg) was orally administered 1 h before injection of compound 48/80. Values are means ± standard error (SE) (*n* = 8). ^#^ *p* < 0.001 *versus* the non-treated control group; * *p* < 0.05, ** *p* < 0.05, *** *p* < 0.01 *versus* compound 48/80 alone group.

Compound 48/80 is a potent activator of connective tissue-type and/or skin mast cells [28]. Therefore, the scratching behavior caused by compound 48/80 may be affected *via* mediators released from mast cells, such as histamine and substance P [29,30]. Therefore, we determined whether luteolin could reduce the scratching behavior and skin vascular permeability induced by pruritogens (compound 48/80 or serotonin) in ICR mice. As shown in Figure 5, compound 48/80 markedly increased the scratching behavior (166.5 ± 16.4 times/60 min) and vascular permeability (100% ± 9.8%) compared with the control (9.8 ± 3.4 times/60 min, 14.5% ± 1.9%; *p* < 0.001).

**Figure 6:**
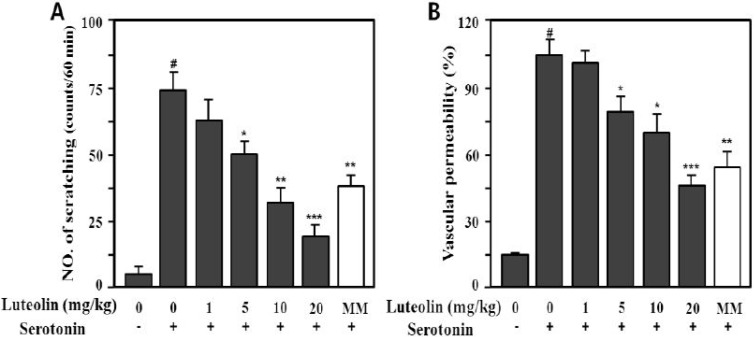
Effect of luteolin on serotonin-induced scratching behavior (**A**) and vascular permeability (**B**) in ICR mice. Scratching behavior was counted for 60 min after intradermal injection of serotonin (100 μg/site). Luteolin (1–20 mg/kg) was administered orally 1 h before serotonin injection. Values are means ± SE (*n* = 8). ^#^ *p* < 0.001 *versus* the non-treated control group; * *p* < 0.05, ** *p* < 0.05, *** *p* < 0.01 *versus* serotonin alone group.

Serotonin (5-HT) also markedly increased the scratching behavior (73.5 ± 10.3 times/min) and vascular permeability (100% ± 9.8%) compared with the control (*p* < 0.001), as shown in Figure 6. Luteolin significantly inhibited compound 48/80- or serotonin-induced scratching behavior at 5, 10 and 20 mg/kg in a dose-dependent manner (*p* < 0.05, *p* < 0.01 and *p* < 0.001, respectively).

Methysergide interacts with serotonin (5-HT2B) receptors. Its therapeutic effect in migraine prophylaxis is associated with antagonism of the 5-HT2B receptor [31] and at a dose of 10 mg/kg has a significantly antagonistic effect on th 5-HT-induced mouse itching response. To investigate the antipruritic effect of luteolin, we used methysergide maleate (MM) as the reference drug. As shown in Figure 5A and Figure 6A, luteolin had a greater antipruritic effect than MM at the same concentration (10 mg/kg).

## 3. Experimental

### 3.1. Chemicals

Iscove’s modified Dulbecco’s medium (IMDM), glutamax-l and fetal bovine serum (FBS) were purchased from GIBCO-BRL, Invitrogen Co. (Grand Island, NY, USA). TNF-α and IL-1β ELISA kits were purchased from R&D Systems (Minneapolis, MN, USA). Histamine ELISA kits were purchased from ALPCO Diagnostics (Salem, NH, USA). Compound 48/80, phorbol 12-myristate 13-acetate (PMA), penicillin/streptomycin (P/S), MM and other reagents were purchased from Sigma-Aldrich (St. Louis, MO, USA).

### 3.2. Perilla Leaves and Luteolin Isolation

Perilla leaves were collected on June 20, 2012 from Aenong Farm (Jinan, Jeollabuk-do, Republic of Korea). The plant was identified and authenticated by Professor Hong-Jun Kim at the College of Oriental Medicine, Woosuk University. A voucher specimen (^#^ML-11-02) was deposited in the author’s laboratory (Professor Seon-Il Jang).

Dried perilla leaves (100 g) were extracted with MeOH (2 L) for 60 min at 25 °C. The MeOH extracts were partitioned with in sequence organic solvents of different polarities to yield *n*-hexane, EtOAc, *n*-BuOH and H_2_O fractions. The EtOAc fraction was subjected to silica gel chromatography with CH_2_Cl_2_–MeOH (lower layers, by volume, 30:1→1:1, 100% MeOH, gradient) as the solvent to yield the perilla luteolin (3',4',5,7-tetrahydroxylflavone). The structure of the compound (Figure 1) was determined by its physicochemical and spectral data (LC-MS, ^1^H- and ^13^C-NMR), which were in agreement with previously reported values [20].

### 3.3. Animals

Male ICR mice (6 weeks old) were purchased from Central Lab Inc. (Seoul, Korea), the Korean branch of Charles River Japan (Kanagawa, Japan). Male Sprague-Dawley rats (8 weeks old) were purchased from Oriental Bio Experimental Center (Gyeanggi-do, Republic of Korea). Animals were maintained in an environmentally controlled housing system and used for experiments after 1 week. The animals were given a standard laboratory diet and water *ad libitum*. All experiments were performed in accordance with Jeonju University Institutional Animal Care and Use Committee guidelines (No. 2012-002).

### 3.4. Cell Culture and Stimulation

HMC-1 cells were cultured in IMDM with glutamax-l supplemented with 10% FBS at 37 °C in a 5% CO_2_ atmosphere at 95% humidity. HMC-1 cells (5 × 10^5^) were treated with 1–20 μM luteolin and then incubated at 37 °C for 8 h. The cell viability was over 95% in all experiments.

### 3.5. MTT Assay

Cell viability was determined by the MTT assay. Briefly, HMC-1 cells (5 × 10^5^/well) were cultured in four-well plates for 8 h after treatment with the various luteolin concentrations. Twenty microliters of MTT solution (5 mg/mL) were added and the cells were incubated at 37 °C for a further 4 h. After washing out the supernatant, the insoluble formazan product was dissolved in DMSO. Next, the optical density at 540 nm in the 96-well culture plates was measured using a microplate reader.

### 3.6. Rat Peritoneal Mast Cell (RPMC) Isolation and Stimulation

The mice were anesthetized, and 10 mL of Tyrode buffer A (10 mM HEPES, 136 mM NaCl, 5 mM KCl, 2 mM CaCl_2_, 2.75 mM MgCl_2_, 5.6 mM glucose, 11 mM NaHCO_3_, 0.56 mM NaH_2_PO_4_ and 0.1% bovine serum) containing 0.1% gelatin were injected into the peritoneal cavity. The cavity was carefully opened, and peritoneal cells were obtained and centrifuged at 150 *×g* for 10 min at room temperature before resuspension in Tyrode buffer A. Mast cells were isolated from the peritoneal cells according to a method described previously [22] and then assessed by toluidine blue staining.

### 3.7. Histamine Assay

RPMCs were resuspended in Tyrode buffer A for treatment with compound 48/80. RPMC suspensions (5 × 10^5^/mL) were preincubated with 1–20 μM luteolin and then stimulated with compound 48/80 (5 μg/mL). Each activated RPMC was centrifuged at 150 *×g* for 10 min at 4 °C and the supernatant obtained. Each supernatant was then assayed for histamine content by ELISA according to the manufacturer’s specifications. Absorption of the avidin-horseradish peroxidase color reaction was measured at 450 nm.

### 3.8. Cytokine ELISA

Secreted cytokine levels in culture supernatants of HMC-1 cells were measured using a sandwich ELISA according to the manufacturer’s protocol (for IL-1β and TNF-α assay, R&D Systems). Absorption of the avidin-horseradish peroxidase color reaction was measured at 450 nm.

### 3.9. Scratching Behavior

Before the experiment, ICR mice were put into acrylic cages (22 cm × 22 cm × 24 cm) for about 10 min for acclimation. Scratching behavior was evaluated as described previously [11,32]. Briefly, luteolin (1–20 mg/kg, body weight) or MM (10 mg/kg) dissolved in PBS was administered orally to the mice. One hour later, compound 48/80 (50 μg/site) or serotonin (100 μg/site) was injected intradermally into the rostral part of the shaved back of the mouse. Normal control mice were injected with the same volume of PBS. Immediately after injection, the mice (one mouse/cage) were placed into an observation chamber and scratching behavior was recorded using a micro camera (ONCCTV, Seoul, Korea). Scratching actions with the hind paw were enumerated for 60 min and recorded for analysis.

### 3.10. Skin Vascular Permeability

The increase in skin vascular permeability caused by scratching agents was assessed as reported previously [33]. After intradermal injection of compound 48/80 (50 μg/site) or serotonin (100 μg/site) into the rostral part of the mouse back, 2% Evans blue solution was injected intravenously into each animal. The animals were sacrificed 60 min later and the scratching agent-injected site (1 × 1 cm) was immediately excised. The skin specimen was dissolved with 1 mL of 1 M KOH solution by overnight incubation and 4 mL of 0.2 M phosphoric acid-acetone mixture were added. After vigorous shaking, the precipitates were filtered and dye was quantified colorimetrically at 620 nm.

### 3.11. Statistical Analysis

Differences among the groups were evaluated by one-way ANOVA, and all values were expressed as means ± standard deviation (SD). Differences among groups were considered significant at *p* < 0.05.

## 4. Conclusions

In conclusion, luteolin from perilla leaves inhibited the production of TNF-α and IL-1β in PMA plus A23187-activated HMC-1cells. Luteolin also suppressed histamine release by compound 48/80-stimulated RPMCs. Furthermore, luteolin reduced the scratching behavior and vascular permeability induced by compound 48/80 or serotonin, thus exhibiting significant anti-inflammatory and antipruritic effects on inflammation and the itching response. Moreover, these results suggest that luteolin has potential as a therapeutic agent for allergic diseases.
